# Serum Alpha-1-Acid Glycoprotein-1 and Urinary Extracellular Vesicle miR-21-5p as Potential Biomarkers of Primary Aldosteronism

**DOI:** 10.3389/fimmu.2021.768734

**Published:** 2021-11-05

**Authors:** Cristian A. Carvajal, Alejandra Tapia-Castillo, Jorge A. Pérez, Carlos E. Fardella

**Affiliations:** ^1^ Department of Endocrinology, School of Medicine, Pontificia Universidad Católica de Chile, Santiago, Chile; ^2^ Department of Endocrinology, Millennium Institute of Immunology and Immunotherapy (IMII-ICM), Santiago, Chile; ^3^ Center for Translational Research in Endocrinology (CETREN-UC), Pontificia Universidad Católica de Chile, Santiago, Chile

**Keywords:** primary aldosteronism (PA), biomarker, lipocalin, miR-21-5p, extracellular vesicles, AGP1, Alpha-1-acid glycoprotein-1

## Abstract

Primary aldosteronism (PA) is the most common cause of secondary hypertension and reaches a prevalence of 6-10%. PA is an endocrine disorder, currently identified as a broad-spectrum phenotype, spanning from normotension to hypertension. In this regard, several studies have made advances in the identification of mediators and novel biomarkers of PA as specific proteins, miRNAs, and lately, extracellular vesicles (EVs) and their cargo.

**Aim:**

To evaluate lipocalins LCN2 and AGP1, and specific urinary EV miR-21-5p and Let-7i-5p as novel biomarkers for PA.

**Subjects and Methods:**

A cross-sectional study was performed in 41 adult subjects classified as normotensive controls (CTL), essential hypertensives (EH), and primary aldosteronism (PA) subjects, who were similar in gender, age, and BMI. Systolic (SBP) and diastolic (DBP) blood pressure, aldosterone, plasma renin activity (PRA), and aldosterone to renin ratio (ARR) were determined. Inflammatory parameters were defined as hs-C-reactive protein (hs-CRP), PAI-1, MMP9, IL6, LCN2, LCN2-MMP9, and AGP1. We isolated urinary EVs (uEVs) and measured two miRNA cargo miR-21-5p and Let-7i-5p by Taqman-qPCR. Statistical analyses as group comparisons were performed by Kruskall-Wallis, and discriminatory analyses by ROC curves were performed with SPSS v21 and Graphpad-Prism v9.

**Results:**

PA and EH subjects have significantly higher SBP and DBP (p <0.05) than the control group. PA subjects have similar hs-CRP, PAI-1, IL-6, MMP9, LCN2, and LCN2-MMP9 but have higher levels of AGP1 (p <0.05) than the CTL&EH group. The concentration and size of uEVs and miRNA Let-7i-5p did not show any difference between groups. In PA, we found significantly lower levels of miR-21-5p than controls (p <0.05). AGP1 was associated with aldosterone, PRA, and ARR. ROC curves detected AUC for AGP1 of 0.90 (IC 95 [0.79 – 1.00], p <0.001), and combination of AGP1 and EV-miR-21-5p showed an AUC of 0.94 (IC 95 [0.85 – 1.00], p<0.001) to discriminate the PA condition from EH and controls.

**Conclusion:**

Serum AGP1 protein was found to be increased, and miR-21-5p in uEVs was decreased in subjects classified as PA. Association of AGP1 with aldosterone, renin activity, and ARR, besides the high discriminatory capacity of AGP1 and uEV-miR-21-5p to identify the PA condition, place both as potential biomarkers of PA.

## Introduction

The etiology of arterial hypertension (AHT) is unknown in more than 80-90% of cases, which is named essential hypertension (EH). One third of EH has been suggested to be associated with endocrine disorders (1). Primary aldosteronism (PA) is an endocrine disorder, currently identified as a broad-spectrum phenotype, spanning from normotension (4% prevalence) to hypertension (10% prevalence) (2–6). PA is characterized by an inappropriately high circulating aldosterone independent of known physiological regulators such as renin, angiotensin II, potassium, and sodium status (e.g., high saline intake) (7). The diagnosis of PA is relevant, not only for its association to high blood pressure but also for the harmful effects in extra-renal tissues, generally associated with the mineralocorticoid receptor (MR) activation by aldosterone which induces inflammation (8, 9), tissue remodeling, and fibrosis (8, 10–14), affecting the renal, heart, the vascular system (endothelial cells and smooth muscles cells), the immune system (15) and the adipose tissue (16).

Several studies have tied to advance in the identification of novel biomarkers for PA that support its early detection and also other reported effects as inflammation, endothelial dysfunction, renal damage, vascular remodeling and (17, 18), and oxidative stress (19, 20). Early “surrogate biomarkers” have been previously evaluated, such as high sensitive C-reactive protein (hs-CRP), Plasminogen inhibitor activator-1 (PAI-1), matrix metallopeptidase 9 (MMP-9) and malondialdehyde (MDA) (8, 9), free Cystatin-C (CysC), and neutrophil gelatinase associated lipocalin (NGAL or LCN2) (21–23). However, none of these biomarkers are currently available in clinical diagnoses for arterial hypertension or PA. Recent proteomic studies have shown that urinary and serum alpha-1-acid glycoprotein-1 (AGP1), also known as ORM1, have been proposed as prognostic biomarkers for inflammatory diseases such as chronic heart failure (24), some types of cancer (25), and lately for PA (26).

Experimental and clinical studies demonstrate that small extracellular vesicles (sEVs) or exosomes are potential biomarkers of disease (27), including in cancer, metabolic disorders, and cardiovascular diseases (28, 29). Urinary EVs originated mainly from cells lining the renal tubules carrying proteins, lipids, RNA, and miRNA, and have been recognized recently as a source of diagnostic biomarkers for different renal and endocrine pathologies (30–36), including primary aldosteronism (26).

MicroRNAs (miRNAs) are short non-coding RNA molecules genome-encoded, that are approximately 22 nucleotides in length and modulate downstream gene expression by post-transcriptional mechanisms, specifically by binding to the 3′ untranslated regions (UTR) of a target messenger RNA (mRNA), leading to mRNA degradation or repression of translation (37–39). Recent literature (30, 37, 40–46) proposes that microRNAs in exosomes are involved in physiological and pathophysiological processes correlated with hypertension (47) response to sodium intake (48) and PA (26, 30). miRNAs are packaged into EVs for transport into different biofluids (e.g., blood, urine) and change according to the metabolic microenvironment (e.g., inflammation) of the parent cell. In endocrine hypertension phenotypes, such as nonclassic-AME (31) and PA, have been identified in the differential expression of EV-associated miRNAs, such as miR-192, miR-204 (31), miRNA-21, and Let-7i. miR-21 has been found in EVs isolated from urine (43), plasma (44), and endothelial cells (45). Romero et al. reported on the protective role of miR-21 in the cardiac pathology triggered by excess aldosterone in the heart of mice and rats (49, 50). Let-7i negatively regulates cardiac inflammation and fibrosis in presence of angiotensin II or aldosterone (49–51). Similarly, Deccman et al. identified circulating miR-30e-5p, miR-30d-5p, miR-223-3p, and miR-7-5p in PA patients with bilateral adrenal hyperplasia (BAH) and aldosterone-producing adenoma (APA). Altogether, these reports highlight the potential role of both miRNA and EV-associated miRNAs as biomarkers or mediators of PA (46).

The current study aimed to evaluate lipocalins LCN2 and AGP1, and specific urinary extracellular vesicles miR-21-5p and Let-7i-5p as novel biomarkers of primary aldosteronism.

## Methods

This study used the serum, plasma, and urine samples from a biobank obtained from a cohort of 206 adult Chilean subjects of both genders, between 18 and 65 years old. The subjects were recruited from outpatient centers associated with the UC-Christus Health Network in Santiago, Chile, following the guidelines of the Declaration of Helsinki and approved by the Ethics Committee of the Faculty of Medicine of the Pontificia Universidad Católica de Chile (Certification CEC-MEDUC 12-207 and 14-268, and updated by CEC-MEDUC 190823001 and 200619004).

All subjects had a sodium diet ad libitum and declared that they did not ingest any herbal products or extreme diets during the month prior to the analysis. Subjects with a BMI >30 kg/m^2^, kidney disease, diabetes mellitus, liver, and heart failure were excluded. Subjects using glucocorticoids, contraceptives, or some interfering drugs, such Ag-II-receptor blockers (ARB), ACE-Inhibitors (ACEI), and spironolactone (MR antagonist), were also excluded.

After exclusion criteria were applied, 132 subjects were included in the study. The subjects were classified as normotensive controls (CTL), have clinical and biochemical parameters in the normal range, essential hypertensives (EH) according to the 2017 ACC/AHA Guidelines for High Blood Pressure (52), and subjects having a positive screening for PA (Aldosterone >9ng/dl, PRA <1 ng/ml*h), according to The Endocrine Society 2016 guidelines (53) and Vaidya et al. (5, 54–56). All studied subjects (PA, EH, and CTL) have a clinical record including medical history and physical examination, as well a biochemical profile, creatinine, electrolytes, aldosterone, plasma renin activity (PRA), serum, and 24-hour and morning urine samples. Aldosterone and PRA were measured by immunoassay using a commercial kit (DiaSorin, Stillwater, MN). Urine samples for uEV isolation were stored at -80C with a 1X protease inhibitor cocktail (Roche, USA).

### Evaluation of the Parameters Associated With Inflammation, Endothelial, and Renal Damage in PA Subjects

The inflammatory status of all subjects was evaluated by measuring hs-CRP with a nephelometric assay (BN ProSpec Systems; Siemens Healthcare Diagnostics Products, Marburg, Germany) and IL-6 by an ELISA with commercial reagents and standards (D6050, R&D Systems, Minneapolis, MN), according to the manufacturer’s protocols. Endothelial damage was evaluated by surrogate markers such as PAI-1, MMP9, and MMP2 activities. PAI-1 was determined by ELISA (HYPHEN BioMed, Neuville sur Oise, France), and MMP9 and MMP2 activities by zymography, as previously described (57). Early renal damage was evaluated with 24-hour urine albuminuria to creatinine ratio (UACR). Albumin is measured by a turbidimetric immunoassay (Roche, Germany), and urine creatinine was measured with a colorimetric assay (Roche, Indianapolis, IN) in a Hitachi Automatic Analyzer 7600 (Roche/Hitachi, Kobe, Japan). Plasma and urinary electrolytes (sodium and potassium) were evaluated with methods previously described (58).

### Determination of Serum Lipocalins AGP1, LCN2, and LCN2-MMP9 in PA Subjects

We measured the serum levels of lipocalins AGP1, LCN2, and LCN2-MMP9 proteins (26) by commercial ELISA immunoassay for AGP1 (Human α1-Acid Glycoprotein Immunoassay, DAGP00, USA R&D Systems, Inc.) according to the manufacturer’s protocol, LCN2 (DLCN20, R&D Systems, Minneapolis, MN), LCN2-MMP9 (DM9L20, R&D Systems, Minneapolis, MN).

### Isolation and Characterization of Urinary Extracellular Vesicles From PA Subjects

Urinary EVs (uEVs) were isolated by a sequential ultracentrifugation protocol previously described by Barros et al. (26). Urinary creatinine was used to normalize samples of uEVs (59, 60). Isolated uEVs were characterized as previously described (26, 31) and according to the International Society for Extracellular Vesicles guidelines (27) using transmission electron microscopy (TEM), nanoparticle tracking analysis (NTA), and western blot with characteristic EV proteins (61).

TEM was performed with 15 μl of uEVs suspension were absorbed onto a 200 mesh carbon-coated copper grid for 1 min. Samples were negatively stained with 2% uranyl acetate solution for 1 min. Grids were visualized in a Phillips Tecnai transmission electron microscope at 80 kV and images were acquired using a SIS-CCD Camera Megaview G2 (62). The concentration and size of uEVs were determined by nanotracking analyses (NTA) performed in a low-volume flow cell (LVFC) of a NanoSight NS300 and NTA 3.2 software (Malvern Instruments Ltd, Malvern, UK). Camera level and detection threshold was optimized to identify individual particles and minimum background noise during recordings (camera level = 12-14; detection threshold = 3-5; flow speed = 50). Particles were tracked by passing a laser beam through the liquid sample and the scattered light was detected and captured in short videos by a sCMOS camera (3 videos of 20 seconds each). The Brownian motion of particles was determined, and the distance moved by the detected particles will be used to calculate the diameter (mean and mode size) and concentration of vesicles using the Stokes-Einstein equation (63).

### Western Blot of Exosome Markers TSG101 and CD9 Proteins

Similar quantities of EVs were resuspended in Laemmli buffer and then separated by SDS polyacrylamide gel electrophoresis (SDS-PAGE) and transferred to nitrocellulose membranes (Bio-Rad, CA, USA), blocked with 5% skim milk in PBS-Tween20 (PBST) 0.1% (vol/vol) for 1 hour and probed with primary rabbit monoclonal anti-TSG101 (1:10.000 Ab125011, Abcam, MS, USA), rabbit monoclonal anti-CD9 (1:500 (D801A) cat#13174; Cell Signaling Technology, MA, USA). After washing, membranes were incubated with horseradish peroxidase-conjugated goat anti-rabbit IgG-HRP (1:10.000; ab6939; Abcam, USA) for 1 hour at RT. Proteins were detected using chemiluminescence (ECL Western Blotting substrate reagent, Pierce, USA) in a Chemi-Doc MP imaging system (Bio-Rad, CA, USA).

### Urinary EV RNA Isolation

RNA from the extracellular vesicle was isolated by organic extraction using the Trizol^®^ reagent according to the manufacturer’s instructions. Two microliters of each RNA sample were pipetted on the NanoQuant Plate™ of the Infinite^®^ M200 PRO spectrophotometer (TECAN; Männedorfl; Switzerland) to quantify the RNA concentration (A260 nm) and purity (A260/A280 nm ratio) using Tecan i-control™ software.

### Expression of miR-21-5p and Let-7i-5p in Urinary Extracellular Vesicles

Reverse transcription of miRNA samples was performed using the TaqMan™ Advanced miRNA cDNA Synthesis Kit (A28007), according to the manufacturer’s instructions. The expression of miRNAs (Hsa-miR-21-5p and Hsa-let-7i-5p) were evaluated with TaqMan™ Advanced miRNA Assay (A25576) and the TaqMan™ Fast Advanced Master Mix (4444557, Applied Biosystems) in the RotorGene 6000 thermocycler (Corbett Research, Sydney, Australia). The amplification reactions were performed as follows: Enzyme activation at 95°C for 20 seconds and 40 cycles of 95°C for 3 seconds, anneal/extend at 60°C for 30 seconds. RNU6 snRNA was used as an internal normalization control (TaqMan™ MicroRNA Assay, ID001973). The fold changes of miRNA expression were calculated using the relative cycle threshold (2−ΔΔCt) method and further normalized by the spot urinary creatinine. Unpaired Kruskal-Wallis test was performed to identify differences in PA patients versus EH and healthy controls.

### Statical Analyses

Clinical, biochemical, and expression data are expressed as median [Q1-Q3]. Data normality was determined by Kolmogorov-Smirnov test. For parametric and non-parametric comparisons between two sets of data, an unpaired Student *t*-test or a Mann-Whitney test were performed. To assess differences between groups of data and an independent variable, a one-way Analysis of Variance (ANOVA) or Kruskal Wallis was performed using a Tukey or Dunn *post hoc* test, respectively. Associations were performed by linear regression by Pearson or Spearman regression according to data normality.

Receiver operating characteristic (ROC) analysis was used to test the ability of lipocalins (LCN2, AGP1) and uEV-associated miRNAs (miR-21-5p and Let-7i-5p) to discriminate PA patients from EH and control subjects. A p value < 0.05 was considered statistically significant. Data were analyzed using GraphPad Prism v9.1 (GraphPad, CA, USA) or SPSS v21 (IBM, USA) software.

## Results

### Clinical and Biochemical Characteristics of Subjects With PA

**Figure 1:**
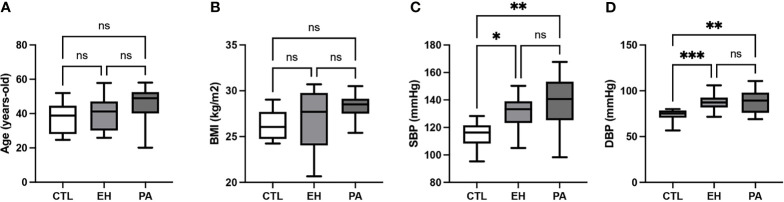
Clinical characteristics of subjects with PA. **(A)** Age (years old). **(B)** Body mass index (BMI; kg/m^2^) **(C)** Systolic blood pressure (SBP; mmHg). SBP was higher in PA and EH subjects in the CTL group. **(D)** Diastolic blood pressure (DBP; mmHg). DBP was higher in PA and EH subjects in the CTL group. Comparison between groups was performed by unpaired one-way ANOVA or Kruskal-Wallis test. Data are presented as median and interquartile range [Q1-Q3], N.S: No significative difference, *p < 0.05, **p < 0.01, ***p < 0.001.

We identified 11 PA subjects (8.3%) in our cohort of study according to the PA criteria described in the Methods section. Clinical and biochemical baseline characteristics are shown in **Table 1**. PA, EH, and CTL groups were similar in age, gender, and body mass index. Systolic (140 [125-153] vs. 134 [123-139] vs 116 [110-121] mmHg, p<0.05) and diastolic blood pressure (89 [76-98] vs. 87 [81-93] vs. 75 [71-78] mmHg, p <0.05) were higher in the PA and EH group compared to healthy controls, respectively (**Figure 1**).

**Table 1:** Clinical and biochemical parameters of subjects identified as control, EH, and primary aldosteronism.

	CONTROL	EH	PA
***N***	13	17	11
**Age (years old)**	37 [28-47]	39 [29-47]	48 [37-53]
**Man (%)**	46	58	55
**BMI (kg/m^2^)**	26.1 [24.7-27.7]	27.7 [24.4-29.7]	28.5 [27.5-29.1]
**SBP (mmHg)**	116 [110-121]	134 [123-139] b	140 [125-153]^a^
**DBP (mmHg)**	75 [71-78]	87 [81-93]	89 [76-98]^a^
**Serum aldosterone (ng/dl)**	9.8 [6.9-12.5]	7.8 [6.1-8.4]	12.7 [10.4-13.7]^a^
Plasma renin activity (ng/mL*h)	1.8 [1.3-2.3]	1.9 [1.4-2.8]	0.8 [0.5-0.9]^a,b ^
**ARR**	5.4 [3.2-7.3]	4.1 [2.6-5.4]	17.9 [13.7-20.8]^a,b^
**Plasma sodium (mEq/l)**	140 [139-141]	141 [140-142]	140 [139-142]
**Plasma potassium (mEq/l)**	4.1 [3.9-4.4]	4.2 [3.8-4.5]	4.2 [3.9-4.4]
**Urinary sodium (mEq/24 h)**	136 [73-202]	162 [114-216]	125 [99-176]
**Urinary potassium (mEq/24 h)**	47 [31-62]	54 [39-66]	53 [41-67]
**Sodium excreted fraction (%)**	0.63 [0.53-0.83]	0.64 [0.34-0.78]	0.57 [0.51-0.88]
**Potassium excreted fraction (%)**	7.2 [5.6-8.2]	7.9 [5.1-9.3]	8.0 [7.4-8.7]

Data are presented as a median and interquartile range [Q1-Q3]. BMI, Body Mass Index; PAS, Systolic Pressure; PAD, Diastolic Pressure; ARR, Aldosterone/Plasmatic Renin Activity Ratio. ^a^Different from the HE group and ^b^the control group. Analysis was performed using Kruskal-Wallis, p < 0.05, and χ^2^ test, p < 0.05.

**Figure 2:**
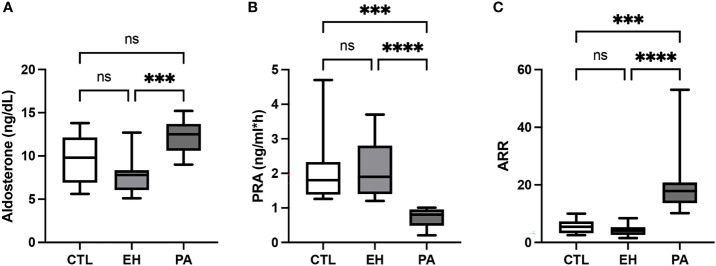
Biochemical characteristics of subjects with PA. **(A)** Serum aldosterone concentration (ng/dL). Serum aldosterone levels were higher in PA subjects in the EH group. **(B)** Plasmatic renin activity (PRA; ng/mL*h). Plasmatic renin activity was lower in PA subjects in both the EH and CTL groups. **(C)** Aldosterone to renin ratio (ARR). ARR was higher in PA subjects in both the EH and CTL groups. Comparison between groups was performed by unpaired one-way ANOVA or Kruskal-Wallis test. Data are presented as median and interquartile range [Q1-Q3], N.S, No significative difference; ***p < 0.001, ****p < 0.0001.

Serum aldosterone was higher in PA in respect to EH, but similar to the control group (12.7 [10.4-13.7] vs. 7.8 [6.1-8.4] vs. 9.8 [6.9-12.5] ng/dL, p <0.0001). PRA was significantly lower in PA in respect to EH and controls (0.8 [0.5-0.9] vs. 1.9 [1.4-2.8] vs. 1.8 [1.3-2.3] ng/mL*h, p <0.0001). The ARR was higher in PA than EH and controls (17.9 [13.7-20.8] vs. 4.1 [2.6-5.4] vs. 5.4 [3.2-7.3], p<0.0001) (**Table 1**, **Figure 2**). No differences were found in plasma and urinary sodium and potassium electrolytes, nor in the fractional excretion of potassium (FEK) or the fractional excretion of sodium (FENa) in PA, EH, and controls (**Table 1**).

### Evaluation of Parameters Associated With Inflammation, Endothelial, and Renal Dysfunction in PA Subjects

**Figure 3:**
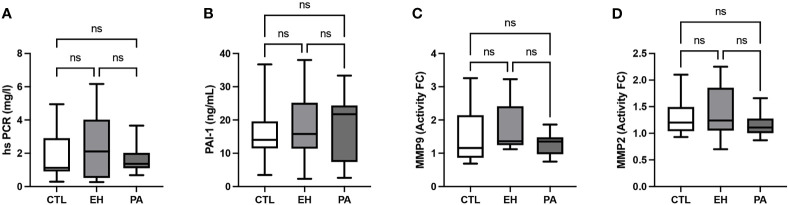
Evaluation of parameters associated with inflammation, endothelial and renal dysfunction in PA subjects. **(A)** High sensitivity C reactive protein (hsPCR; mg/L). **(B)** Plasminogen activator inhibitor – 1 (PAI-1; ng/mL). **(C)** Metalloproteinase 9 (fold change activity). **(D)** Metalloproteinase 2 (fold change activity). No differences of parameters associated with inflammation, endothelial and renal dysfunction were found between groups. Comparison between groups was performed by unpaired one-way ANOVA or Kruskal-Wallis test. Data are presented as a median and interquartile range [Q1-Q3], N.S, No significative difference.

We found similar plasma levels of hs-CRP (1.4 [1.1-2.0] vs 2.1 [0.5-4.0] vs 1.1 [0.9-2.9] mg/L, p NS) and Interleukin 6 (IL-6) (3.0 [1.5-3.1] vs. 3.2 [2.7-3.9] vs. 3.0 [1.7-3.2] pg/ml, p NS) in PA from those found in EH and controls, respectively. Endothelial markers PAI-1, MMP9 and MMP2 were also evaluated, showing no differences in PA respect to EH or control subjects (**Table 2**, **Figure 3**). Renal dysfunction was evaluated with the urinary albumin to creatinine ratio (UACR) (3.2 [2.0-4.6] vs. 3.6 [1.5-5.4] vs. 4.3 [1.9-6.6] mg/gr Crea) which was also similar in PA and EH and Controls (**Table 2**).

**Table 2:** Evaluation of parameters associated with inflammation, endothelial and renal damage in PA subjects, EH, and controls.

	CONTROL	EH	PA
**Hs-CRP (mg/l)**	1.1 [0.9-2.9]	2.1 [0.5-4.0]	1.4 [1.1-2.0]
**IL-6 (pg/ml)**	3.0 [1.7-3.2]	3.2 [2.7-3.9]	3.0 [1.5-3.1]
**PAI-1 (ng/ml)**	14.0 [11.5-19.5]	15.8 [11.4-21.2]	21.1 [7.3-24.4]
**MMP9 (activity FC)**	1.2 [0.8-2.2]	1.4 [1.2-2.4]	1.4 [1.0-1.5]
**MMP2 (activity FC)**	1.2 [1.0-1.5]	1.2 [1.0-1.9]	1.1 [1.0-1.3]
**Urinary albumin (mg/g creatinine)**	4.3 [1.9-6.6]	3.6 [1.5-5.4]	3.2 [2.0-4.6]

hs-PCR, High sensitivity C reactive protein; IL-6, Interleukin-6; PAI-1, Plasminogen activator inhibitor-1; MMP9, Matrix metalloproteinase-9 activity (fold change); MMP2, Matrix metalloproteinase-2 activity (fold change); LCN2, Serum LCN2 concentration; LCN2-MMP9, Serum LCN2-MMP9 concentration; LCN2+MMP9, Serum LCN2+LCN2-MMP9 concentration; AGP1, Serum AGP1 concentration. Data are presented as a median and interquartile range [Q1-Q3]. Statistical analyses were performed using Kruskal-Wallis (Dunn´s) with significance p < 0.05.

### Determination of Serum AGP1, LCN2, and LCN2-MMP9 in PA Subjects

**Figure 4:**
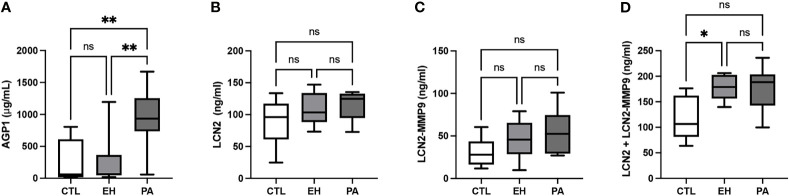
Determination of serum AGP1, LCN2, LCN2-MMP9, and in PA subjects. **(A)** Serum AGP1 concentration (µg/mL). We detected higher levels of AGP1 in PA subjects in both EH and CTL groups. **(B)** Serum LCN2 concentration (ng/mL). LCN2 concentration was similar between groups **(C)** Serum LCN2-MMP9 concentration. LCN2-MMP9 concentration was similar between groups (ng/mL). **(D)** Serum LCN2 + LCN2-MMP9 concentration (ng/mL). Serum levels of LCN2 + LCN2-MMP9 were higher in EH subjects in the CTL group. LCN2 + LCN2-MMP9 concentration was similar between PA and EH subjects and PA and CTL subjects. Comparison between groups was performed by unpaired one-way ANOVA or Kruskal-Wallis test. Data are presented as a median and interquartile range [Q1-Q3]. N.S, No significative difference, *p < 0.05, **p < 0.01.

**Figure 5:**
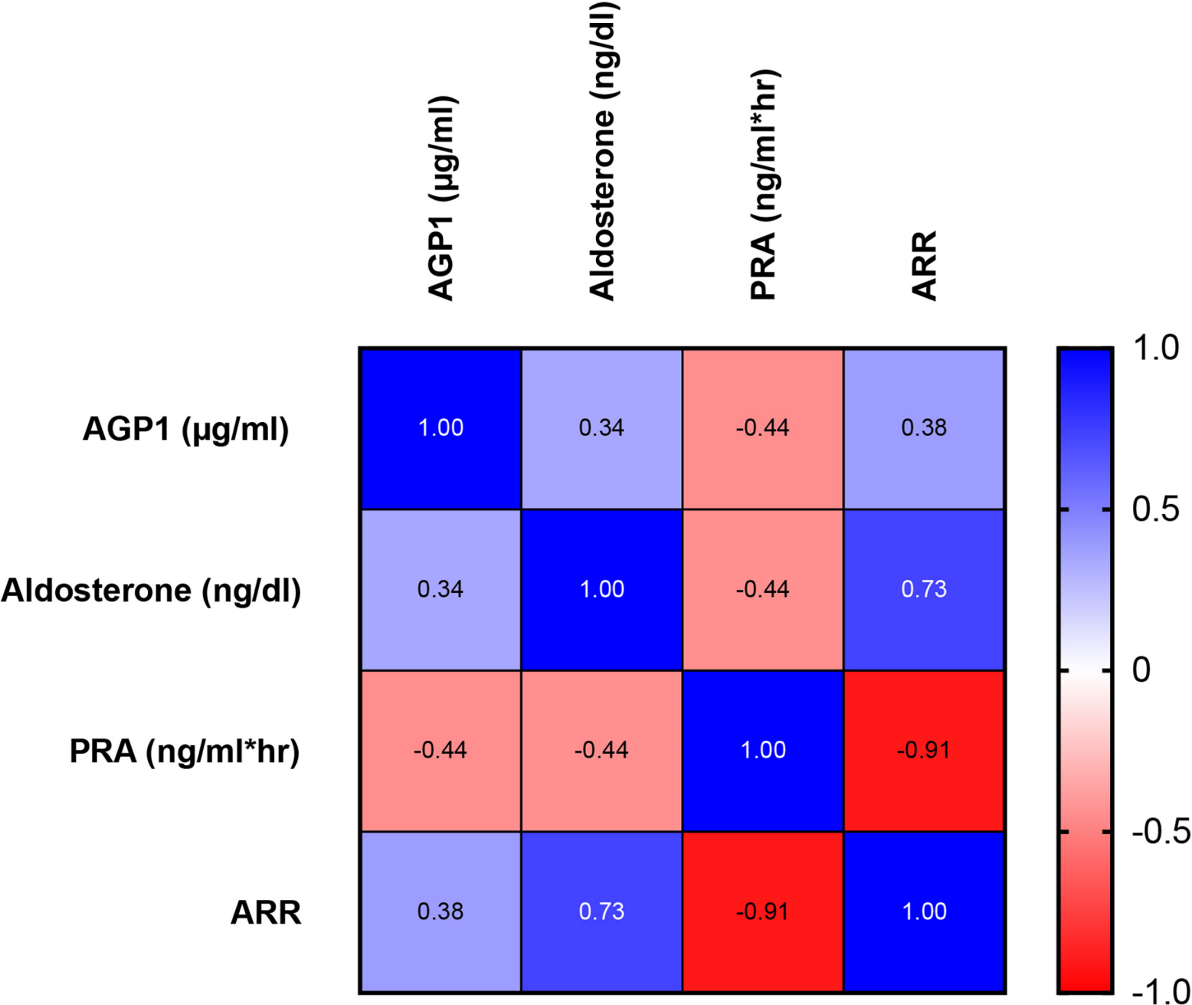
Heat map of AGP1 associations with serum Aldosterone, PRA, and ARR in PA, EH, and CTL subjects. Positive associations are presented in blue gradient with the respective ρ (rho) value. Similarly, negative associations are presented in the red gradient. We observed a significant association between AGP1 concentration and the 3 relevant biochemical parameters in primary aldosteronism screening. Association studies were performed by Spearman test, p < 0.05.

We detected higher levels of AGP1 in PA (934.1 [736.5-1255] vs 62.50 [47.1-365.9] and 60.7 [18,0-609,0] ug/ml, p<0.01) compared to EH and controls subjects. LCN2 and LCN2-MMP9 were similar between the groups (p NS) (**Table 3**). Total LCN2 was found to be higher in EH with respect to the control group, meanwhile, PA was similar to EH but did not reach a significant difference when compared to the control (**Figure 4**). We observed significant associations of AGP1 with Aldosterone (rho = 0.34, p <0.05), with PRA (rho = -0.44, p <0.01) and with ARR (rho = 0.38; p <0.05) (**Figure 5**).

**Table 3:** Determination of serum AGP1A, LCN2, and LCN2-MMP9 in PA subjects.

	CONTROL	EH	PA
**AGP1 (mg/ml)**	60.7 [18-609]	62.5 [47.1-365.9]^a,c^	934.1 [736.5-1255]^a,b^
**LCN2 (ng/ml)**	96 [61-117]	104 [88-133]	123 [80-131]
**LCN2-MMP9 (ng/ml)**	28 [16-43]	45 [29-65]	52 [29-75]
**Total LCN2 (ng/ml)**	107 [81-162]	179 [156-202]^c^	190 [172-214]^b^

AGP1, Serum AGP1 concentration. LCN2, Serum LCN2 concentration; LCN2-MMP9, Serum LCN2-MMP9 concentration; Total LCN2, sum of free LCN2 and LCN2-MMP9 complex. Data are presented as a median and interquartile range [Q1-Q3]. Statistical analyses were performed using Kruskal-Wallis (Dunn´s) with significance p <0.05. ^a^PA different from the EH group, ^b^PA different from the control group, and ^c^EH different from the control group.

### Characterization and Quantification of Urinary Extracellular Vesicles

**Figure 6:**
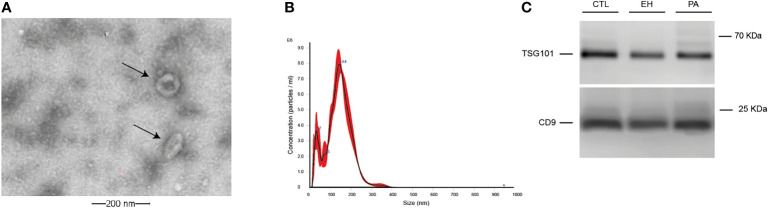
Characterization and quantification of urinary EVs. **(A)**. Identification of uEVs by Transmission Electron Microscopy (TEM) (indicated by black arrows). **(B)** Representative size distribution plot from uEVs using a NanoSight NS300 instrument. **(C)**. Western blot of classic extracellular vesicles markers TSG101 and CD9.

**Figure 7:**
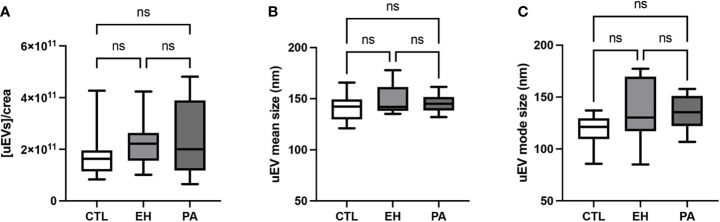
Quantification of uEVs by NTA. **(A)** Urinary creatinine normalized uEVs concentration (uEVs particles/mg creatinine). **(B)** Mean diameter of uEVs particle size distribution (nm). **(C)** Mode diameter of uEVs particle size distribution (nm). uEVs concentration and diameter were similar between groups. Comparison between groups was performed by unpaired one-way ANOVA or Kruskal-Wallis test. Data are presented as a median and interquartile range [Q1-Q3]. N.S: No significant difference.

We isolated uEVs from all subjects in this study. **Figure 6** shows a representative image of isolated uEVs with a donut-shape morphology by TEM (**Figure 6A**), a characteristic plot size/concentration from NTA with the main peak near to 150 nm (**Figure 6B**), and the western-blot of EV markers CD9 and TSG101 (**Figure 6C**). No differences were observed in concentration, mean and mode size of uEVs measured by NTA in PA, EH, and controls (**Table 4** and **Figure 7**).

**Table 4:** Characterization by NTA of urinary extracellular vesicles.

	CONTROL	EH	PA
***uEV (particle/g crea)***	1.63x10^11^ [1.14 x10^11^-1.95 x10^11^]	2.21 x10^11^ [1.55 x10^11^-2.63 x10^11^]	2.0 x10^11^ [1.18 x10^11^- 3.89 x10^11^]
**uEV mean size (nm)**	142 [129-149]	141 [138-161]	145 [139-152]
**uEV mode size (nm)**	121 [109-129]	130 [117-169]	135 [122-155]

Statistical analyses were performed using Kruskal-Wallis (Dunn´s) with significance p <0.05.

### Expression of miR-21-5p and Let-7i-5p in Urinary Extracellular Vesicles

**Figure 8:**
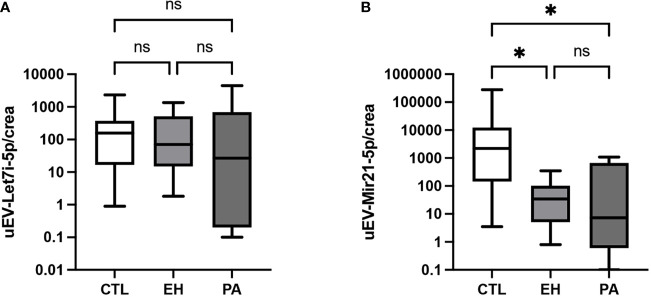
Expression of miR-21-5p and Let-7i-5p in uEVs. **(A)** miR-Let7i-5p expression in uEVs normalized by urinary creatinine (RU/mg creatinine). No differences in miR-Let7i-5p levels were found between groups. **(B)** miR-21-5p expression in uEVs normalized by urinary creatinine (RU/mg creatinine). uEVs miR-21-5p expression was higher in PA and EH subjects respect CTL group. Comparison between groups was performed by unpaired one-way ANOVA or Kruskal-Wallis test. Data are presented as a median and interquartile range [Q1-Q3]. N.S: No significative difference, *p < 0.05, **p < 0.01.

We identified a low expression of miR-21-5p in uEVs in PA and EH in the control group. No difference was detected of miR-21-5p between the PA and EH group (**Table 5**). Concerning Let-7i-5p, we did not detect any differences between all groups (**Table 5** and **Figure 8**).

**Table 5:** Expression of miR-21-5p and Let-7i-5p in urinary extracellular vesicles.

	CONTROL	EH	PA
**miR-21-5p (RU/Crea)**	2194 [143.5-12311]	34.1 [5.1-101.7]^c^	7.3 [0.6-667.5]^b^
**Let7i-5p (RU/Crea)**	157.2 [16.7- 374.5]	70.1 [14.9 -515.4]	26.7 [0.2-684.9]

RU/Crea, Relative units/mg creatinine. Statistical analyses were performed using Kruskal-Wallis (Dunn´s) with significance p <0.05. ^b^PA different from the control group, and ^c^EH different from the control group.

### Receiver Operating Characteristic Curve Analyses for AGP1 and miR-21-5p

**Figure 9:**
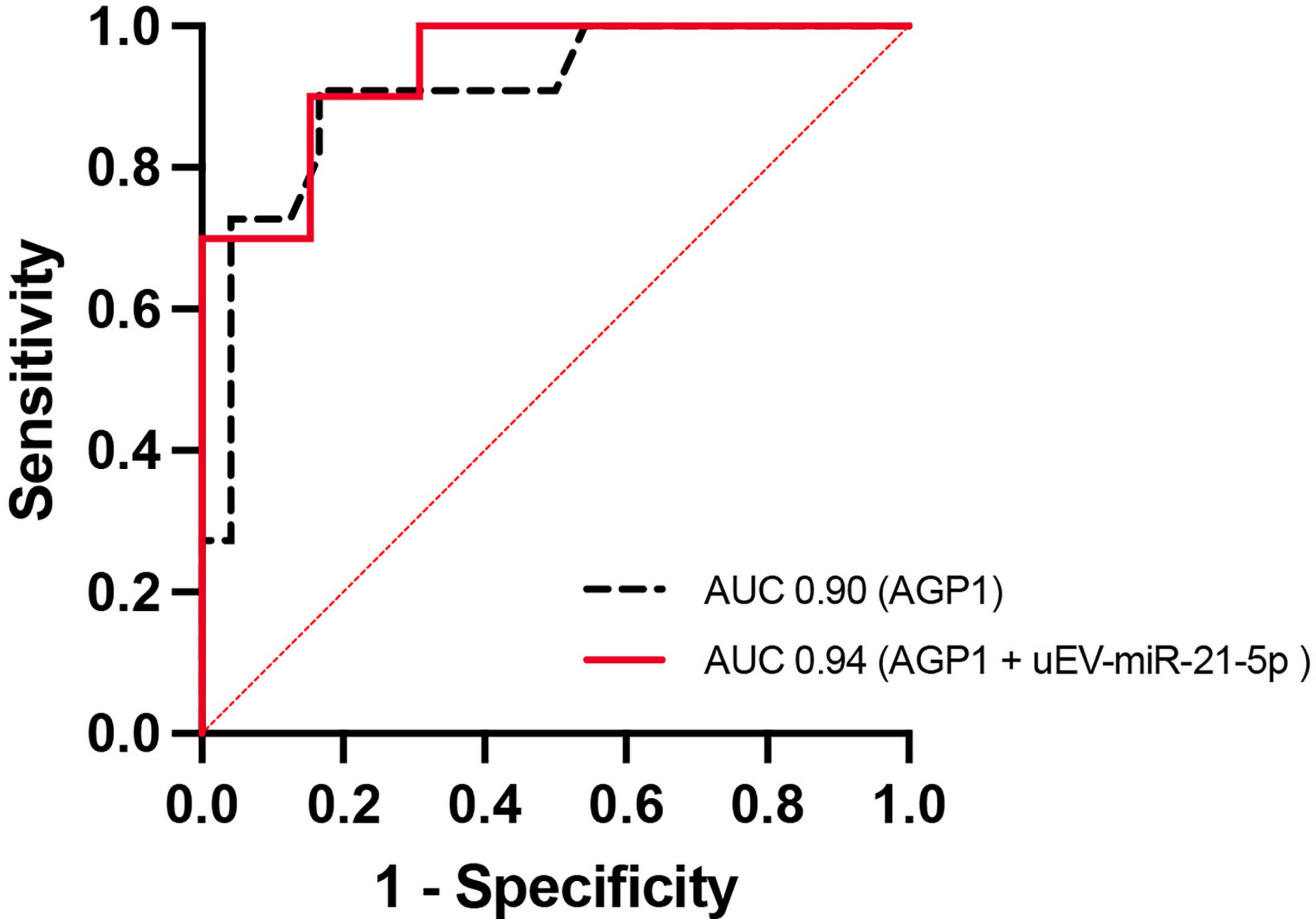
Regression model and Receiver operating characteristic (ROC) curve. ROC curve for serum AGP1 levels (black) and serum AGP1 levels + uEVs associated miR-21-5p (red) can discriminate the PA condition from EH and CTL groups.

Receiver operating characteristic (ROC) analysis showed that a serum AGP1 concentration of 647.9 mg/ml had the best sensitivity (90%) and specificity (83%) to discriminate PA from EH and control subjects. In this analysis, the area under the curve (AUC) for AGP1 was 0.90 (IC 95 [0.79 – 1.00], p <0.001) (**Figure 9**) and for miR-21-5p (AUC 0.63 [0.40-0.86], p NS]. The ROC curve for both AGP1 + EV-miR-21-5p showed a sensitivity of 90% and specificity of 85% with an AUC of 0.94 (IC 95 [0.85 – 1.00], p<0.001) (**Figure 9**).

## Discussion

In the present study, we evaluated the concentration of lipocalins AGP1 and LCN2, and the expression of miR-21-5p and Let-7i-5p in uEVs as potential biomarkers of PA. We observed a higher concentration of AGP1 in PA subjects, which is associated with the critical variables used to screen PA, as plasma aldosterone, renin, and ARR. Further to these novel findings, we noted a low expression of miR-21-5p in PA subjects, which is interesting since it supports a combinate model for the identification of PA conditions. We suggest that both AGP1 and miR-21-5p are associated with the pathogenic course of the primary aldosteronism and can be useful in the design of a novel diagnostic algorithm for PA. There is also a widely accepted consensus regarding a positive screening for PA is an ARR >30 ng/dL per ng/mL/h, with suppressed renin (PRA<1.0 ng/mL*h or DRC <10 uUI/ml) and an aldosterone concentration >15 ng/dL. Some studies have been identified that can improve the detection of milder forms of primary aldosteronism when using less conservative ARR thresholds with suppressed renin activity and plasma aldosterone levels >9 ng/dL (5, 53, 54), which is in agreement with the outcome of this study.

We found similar levels of hs-CRP and IL-6 as markers of inflammation in PA subjects, which were similar to EH and control groups, according to previous studies (12, 64). Similarly, endothelial damage markers (PAI-1, MMP9, and MMP2) and renal function markers (Urinary albumin (UACR)) do not show any significant changes in PA compared with EH and controls. It suggests these subjects, currently classified as subclinical PA (54, 65) do not have chronic inflammation, vascular compromise, or renal function impairment as is seen in overt or classic PA. Hence, is highly necessary novel and sensitive biomarkers aimed to detect subclinical PA and avoid complications associated with the renal and extra-renal effects reported in classic PA.

This perspective is the first to report findings that show a higher serum AGP1 concentration in PA than EH and controls subjects (**Figure 4**). We also observed a significant association of AGP1 with classic screening parameters for PA (e.g., aldosterone, PRA, and ARR) (**Figure 5**). Moreover, we found by discriminative analyses by ROC curves that AGP1 can identify PA from HE&CTL subjects with high sensitivity and specificity. All these results suggest that circulating AGP1 protein is a novel and potential biomarker of PA, which was also suggested for AGP1 protein in urinary exosomes (26). Since AGP1 is a protein from the family lipocalin associated with the acute phase response with immunomodulatory properties (66, 67), affected by glucocorticoids (68–70) and mineralocorticoids (71), we suggest that AGP1 expression is modified by high aldosterone levels through MR activation, having a dual role as a potential biomarker of PA, and possible mediator of the tissue response to high aldosterone. Further clinical and animal model or *in-vitro* studies using MR antagonists should be performed to support this hypothesis.

Similarly, we measured free LCN2 and LCN2 conjugated with matrix metalloproteinase 9 protein as a potential biomarker of PA (72). We found an increase only in total LCN2 (the sum of free and complexed LCN2) in EH, but it did not reach a significant difference in PA when compared with the control or EH. LCN2 is a proinflammatory molecule upregulated in obese individuals or patients with cardiometabolic syndrome, as also has been described in classic PA (73, 74) and is suggested as an MR sensitive protein (75, 76). LCN2 expression is influenced by several factors including obesity, salt intake, aging, infection, and inflammatory status (72, 74, 77–79). Since these subjects have a middle or subclinical PA, with no clear evidence of inflammation, renal/vascular damage (**Figure 3**), or concomitant cardiometabolic disease, we hypothesize the LCN2 fails to increase in these PA subjects since they require a concomitant hit as inflammation (78, 79), obesity (high adipose tissue) (74), or high salt intake (72) to increase the circulating LCN2 levels.

We studied the urinary extracellular vesicles as a source of potential biomarkers for PA (26, 30). In the present study, uEVs showed similar particle concentration and size in PA subjects with respect to EH and controls (**Table 4**). Previous studies in PA show similar findings in uEVs concentration (26) but differ from studies in circulating EVs in PA (75, 76), where they reported an increased concentration of circulating EVs in the serum of PA patients when compared with essential hypertensives and attributed it to an enhanced biological response of the endothelium to aldosterone *in vivo* (75), which has also been observed *in vitro* (80–82). These differences could be related to the PA classification, overt PA versus subclinical PA, and also the different biofluids used to quantify the impact of high aldosterone in EV concentration, serum versus urine, which is associated with distinct mechanisms and the rates of EV shedding that have different tissues (e.g., vascular endothelium vs renal epithelia).

Based on previous reports, we measured the expression of two miRNA in urinary EVs, miR-21-5p, and Let-7i-5p, as potential biomarkers of PA. We observed that uEV-associated miR-21-5p expression in uEVs from PA were lower than controls (**Figure 8**) and similar to EH, however a trend to lower levels was observed in PA. This result suggests that uEV-miR-21-5p is downregulated and associated with pathophysiological mechanisms depending on both high BP and PA conditions. miR-21-5p expression is regulated by cytokines, inflammatory modulators (e.g., NF1, AP1), and steroids. Downregulation of miR-21-5p would affect the downstream target genes related with inflammation (83) as IL-1B gene, aldosterone effect as NEDD4, YWHAZ, SCL12A2 genes, and fibrotic processes (42, 84) as COL1A and COL4A1 genes (**Table 6**). Prospective animal models and *in vitro* studies with miR-21-5p are necessary to gain depth of understanding about the role of this miRNA in high aldosterone conditions in renal epithelia, as occurs in PA.

**Table 6:** Target genes of miR-21-5p and Let-7i-5p, biological process associate and its predicted renal and global effect.

miRNA	Gene target	Biological process	Predicted effect	Global effect
**Hsa-miR-21-5p**	IL1B IL12A IL10	- regulation of lymphocyte mediated immunity - regulation of adaptive immune response	promote an inflammatory state characterized by vascular infiltration of immune cells	Increase inflammation
	COL10A1 COL12A1 COL13A1 COL1A1 COL4A1	- collagen catabolic process - extracellular matrix disassembly	degradation and reorganization of extracellular matrix scaffold	Hypertrophy or hyperplasia of cardiac myocytes and vascular smooth muscle cells (VSMCs)
	NEDD4	protein polyubiquitination	Regulates ENaC function by controlling the number of channels at the cell surface	Increase plasma volume
	SLC12A2	- Mediates sodium and chloride reabsorption. - Plays a vital role in the regulation of ionic balance and cell volume	Increased renal Na+ reabsorption	Increase plasma volume
	TIAM1	GEFs mediate the exchange of guanosine diphosphate (GDP) for guanosine triphosphate (GTP).	Regulator involved in the activation of Rac1 induced by salt loading and aldosterone.	Salt sensitive hypertension
	YWHAZ	positive regulation of signal transduction by binding to phosphoserine-containing proteins	14-3-3 proteins modulate the expression of epithelial Na+ channels	Increase plasma volume
**Hsa-let-7i-5p**	TGFBR1	Is a multifunctional cytokine affecting many cell types and tissue remodeling processes, including angiogenesis and organ fibrosis. TGF-β mediates tissue fibrosis associated with inflammation and tissue injury.	TGF-β increased fibroblast activation, proliferation, and excessive extracellular matrix (ECM) production	increased fibroblast activation, proliferation, and excessive ECM production. Increase fibrosis
	AQP2	renal water homeostasis	increasing the retention of water and sodium	Increase plasma volume
	COL1A1 COL1A2 COL24A1 COL3A1	extracellular matrix organization	degradation and reorganization of extracellular matrix scaffold	hypertrophy or hyperplasia of cardiac myocytes and vascular smooth muscle cells (VSMCs)
	DNMT3A DNMT3B	- DNA methylation on cytosine within a CG sequence - S-adenosylmethioninamine metabolic process - methylation-dependent chromatin silencing - regulation of gene expression by genetic imprinting	Increased promoter methylation of HSD11B2 gene	Decreased cortisol to cortisone metabolism; High F/E ratio
	IL10 IL12A IL13 IL15 IL17RA IL6 IL6R IL8	- positive regulation of cytokine production - inflammatory response	promote an inflammatory state characterized by vascular infiltration of immune cells	Increase inflammation
	NEDD4	protein polyubiquitination	Regulates ENaC function by controlling the number of channels at the cell surface	Increase plasma volume
	ORM1 ORM2	- acute-phase response - response to stress	Functions as transport protein in the blood stream.	Increase due to acute inflammation
	SCNN1A	- sodium ion homeostasis	Increased renal Na+ reabsorption	Increase plasma volume
	SLC12A1	- It plays a key role in concentrating urine and accounts for most of the NaCl resorption	Increased renal Na+ reabsorption	Increase plasma volume
	YWHAZ YWHAE	- mediate signal transduction by binding to phosphoserine-containing proteins.	14-3-3 proteins modulate the expression of epithelial Na+ channels	Increase plasma volume

Gene target identification for identified miRNAs was performed using 5 miRNA gene target databases: miRmap, miRWalk, TargetScan, miRanda, and RNA22.

With respect to uEV-associated Let-7i-5p, we did not observe any differences in Let-7i-5p expression in all groups. Let-7i has been found in either urine (31) and plasma exosomes (44) and is associated with RAAS, mediating inflammation and fibrosis, in both *in vitro* models and experimental models of kidney disease (51, 85). Let-7i regulates downstream target genes TGFBR1, IL6, IL10, COL1A1, COL3A1, DNMT3A, NEDD4, ORM1, VIM, FN1, ACTIN, SCL12A1, and YWHAZ, among others (85–87) (**Table 6**). In the current study, we did not find differences in inflammation parameters, and were unable to measure other important parameters related to fibrosis in these PA subjects, such as the procollagen type 1 protein (PINP, COL1A1).

The ROC curves analyses with AGP1 and miR-21-5p as significant variables associated with PA subjects, support a simple (AGP1) or combinate model (AGP1 + miR-21-5p) to discriminate PA with significant AUC of 90% or 94%, respectively. This AUC is similar to previous reports on AGP1 in uEVs (92%), which support free or uEV-associated AGP1 as potential biomarkers of PA (26).

In summary, we found higher levels of serum AGP1 and lower uEV-miR-21-5p expression in subjects classified as PA with respect to EH and controls. Besides the high discriminatory capacity identified by ROC curves, the association of AGP1 with aldosterone, PRA, and ARR, place both as potential biomarkers of PA. Further studies examining the possible role of AGP1 and miR-21-5p as a mediator of the pathogenic course of PA are encouraged.

## Data Availability Statement

The raw data supporting the conclusions of this article will be made available by the authors, without undue reservation.

## Ethics Statement

The studies involving human participants were reviewed and approved by Unidad de Ética y Seguridad de la Investigación, CEC-SaludUC, Pontificia Universidad Católica de Chile. The patients/participants provided their written informed consent to participate in this study.

## Author Contributions

CC, AT-C, and CF contributed to conception and design of the study. CC and CF provide with the contribution of patients or study material. CC, AT-C, JP performed the collection and/or obtaining of results, organized the database, and performed the statistical analysis. CC and AT-C achieve the analysis and interpretation of the results. CC wrote the first draft of the manuscript. All authors contributed to manuscript revision, read, and approved the submitted version.

## Funding

This study was supported partially by grants ANID-FONDECYT 1212006 (CC) and 3200646 (AT-C); CONICYT-FONDEQUIP EQM150023 (CC); ANID–Millennium Science Initiative Program- IMII P09/016-F, ICN09_016 (CF); SOCHED 2019-09 (CC).

## Conflict of Interest

The authors declare that the research was conducted in the absence of any commercial or financial relationships that could be construed as a potential conflict of interest.

## Publisher’s Note

All claims expressed in this article are solely those of the authors and do not necessarily represent those of their affiliated organizations, or those of the publisher, the editors and the reviewers. Any product that may be evaluated in this article, or claim that may be made by its manufacturer, is not guaranteed or endorsed by the publisher.
